# Associations between reading and writing postures and myopia among school students in Ningbo, China

**DOI:** 10.3389/fpubh.2022.713377

**Published:** 2022-08-12

**Authors:** Danjie Jiang, Bijun Shi, Hua Gao, Yanbo Guo, Shaoying Zhou, Yan Zhang

**Affiliations:** Ningbo Municipal Center for Disease Control and Prevention, Ningbo, China

**Keywords:** reading and writing posture, myopia, school students, public health, near work

## Abstract

**Background:**

We conducted this study to investigate the prevalence of myopia among school students in Ningbo and to explore the associations between reading and writing postures and myopia.

**Methods:**

A population-based and cross-sectional study was conducted, and 3,256 school students aged 8–19 years were recruited. Each enrolled subject was assessed for uncorrected distance visual acuity (UDVA) using a standard logarithmic visual acuity E-chart and a non-cycloplegic autorefraction examination. Self-administered questionnaires were used to investigate myopia-related reading and writing postures and behavioral habits among school students.

**Results:**

The prevalence of myopia among primary school, middle school, and high school students was 61.49, 81.43, and 89.72%, respectively. Regarding the associations between reading and writing postures and myopia, we identified that a reading distance >33 cm is a protective factor for myopia in female students [odds ratio (OR) = 0.31, 95% confidence interval (CI) = 0.15–0.64], in both primary school (OR = 0.55, 95% CI = 0.30–0.99) and middle school (OR = 0.37, 95% CI = 0.15–0.90).

**Conclusions:**

A reading distance >33 cm can be used as an additional measure to prevent and control myopia. Proper postural measures for reading and writing may have educational and public health benefits.

## Introduction

In recent decades, myopia in children and adolescents has become a major public health problem (1). In addition to genetic factors (2), environmental factors and habits and customs play an important role in the onset and development of myopia in children and adolescents, such as higher educational attainment and school achievement (3), a greater amount of near work (4), body stature (5), degree of urbanization (6), and degree of outdoor activity (7). The study and control of environmental factors are currently the focus of myopia prevention. Among them, reading and writing posture-related near vision behavior is one of the focal points of intervention (8).

To supervise and correct children's bad writing posture at any time, there is “one Chi, one fist, and one Cun” principle in China (Chi and Cun are units of measurement in ancient China, one Chi = 33 cm, one Cun = 3.3 cm, one fist is the width of a fist), and the distance between the eyes and the book should be about 33 cm, the distance between the chest and the desk should be about the width of a fist, and the distance between the fingers holding the pen and pen tip should be about 3.3 cm. In China, almost all of the criteria for reading and writing postures are based on the “three ones” principle. Some studies further refined or supplemented the abovementioned criteria, and a few studies adopted only one of them (9). The distance between the eyes and the book is the most commonly used, and the standard of judgment is usually 30–33 cm. Other research-related standards mainly include reading and writing distance, short-distance reading time, determining whether the body is sitting upright, and determining whether there is a forward or backward skew.

Despite several decades of research, the role of reading postures and near work in myopia remains conflicting, with some studies reporting no relationship and other studies finding the opposite (9). Rather than the daily duration of near work, there has been increasing interest in absolute working distance and duration of continuous near vision. Several studies found that shorter working distances (<30 cm) and continuous near-work activity (>30 min) are risk factors for the onset and progression of myopia. For example, in a population study in Canada, the refraction became more myopic by 0.43 and 0.30 D with an increase in near work by every hour after controlling for age, gender, and education of participants aged 5–14 and 15–30 years, respectively (10). Mavrakanas et al. conducted research on 1,738 Greek high school students aged 15–18 years and found that a significantly higher proportion of students with myopia studied 5 h/days more than students with no myopia (43.14 vs. 28.62%, *p* < 0.001) (11). In an Australian population-based study, Ip et al. reported that close reading distance (<30 cm) and continuous reading (>30 min) independently increased the odds of having myopia (12).

We conducted this study to investigate the prevalence of myopia among school students in Ningbo and the associations between reading and writing postures and myopia.

## Methods

### Study population

A population-based and cross-sectional study was conducted to investigate the associations between reading and writing postures and myopia in Ningbo, Zhejiang. Participants were selected using a complex, stratified, multistage sample design. We randomly selected one urban area and one suburban county in Ningbo, with seven schools (two primary schools, two middle schools, two high schools, and one vocational high school) randomly selected in the urban area and five schools (two primary schools, two middle schools, and one high school) randomly selected in the suburban county. Investigations were conducted on whole classes at each grade level in primary, middle, and high schools, with at least 80 students selected from each grade. A total of 3,256 school students aged 8–19 years were recruited for our study, of whom 1,088 were primary school students, 1,088 were middle school students, and 1,080 were high school students.

Informed consent was obtained from all participants. The study was approved by the ethics committee of Ningbo Municipal Center for Disease Control and Prevention and followed the tenets of the Declaration of Helsinki.

### Ocular measurements

Ocular measurements included distance vision examinations and refraction tests. The staff consisted of at least one specialist ophthalmologist and several technicians or nurses in specialist areas. All testers were trained to be proficient in the testing methods and could only start work after passing the test. Uncorrected distance visual acuity (UDVA) was uniformly performed using a standard logarithmic visual acuity E-chart, and the test results were recorded using the five-point recording method. Non-cycloplegic autorefraction examinations were conducted using Topcon RM-800 computer optometry (Topcon Co., Japan) to read the values of spherical lenses, cylinder, and axial length. Spherical equivalent (SE) was calculated as spherical lenses plus 1/2 cylinder. Myopia was defined as UDVA <5.0 and SE < −0.50D. Subjects wearing keratoconus lenses were excluded.

### Questionnaire study

Self-administered questionnaires, including students' basic information, myopia-related reading and writing postures, and behavioral habits, were used. After the unified training, investigators sent questionnaires to schools, asked the students to fill in, and requested the teachers to collect them back. After collecting and reviewing the questionnaires, in case of incomplete and illogical questionnaires, investigators contacted the respondent to explain it and refill the questionnaire.

### Statistical analysis

The survey data were entered into the EpiData 3.1 database. After the logical check and data check, statistical analysis was performed using SPSS 20.0 software. Participants' characteristics were described using means and standard errors for continuous variables, and numbers and percentages for categorical variables. To determine the associations between reading and writing postures and myopia, we applied logistic regression analysis to different gender groups and different school-type groups. The regression model was adjusted according to age, gender, and grade. A *p*-value < 0.05 was considered to be statistically significant.

## Results

The main characteristics of the study participants are reported in Table 1. Of the 3,256 school students, 33.42% were primary school students, 33.42% middle school students, and 33.17% high school students. The average age of the three groups was 10.66, 13.59, and 16.59 years. The proportion of male students in the three groups was 53.77, 50.37, and 46.20%, respectively. The situation of reading and writing postures is also shown in Table 1.

**Table 1:** Characteristics of all subjects included in the study.

**Characteristics**	**Primary School** **(*N =* 1,088)**	**Middle School** **(*N =* 1,088)**	**High School** **(*N =* 1,080)**
Age	10.66 ± 0.86	13.59 ± 0.92	16.59 ± 0.89
Gender (M/F)	585/503	548/540	499/581
**Myopia prevalence**	61.49% (669/1,088)	81.43% (886/1,088)	89.72% (969/1,080)
Male	58.63% (343/585)	78.28% (429/548)	87.17% (435/499)
Female	64.81% (326/503)	84.63% (457/540)	91.91% (534/581)
**When you're reading and writing**
**The chest is more than the width of a fist from the edge of the table**			
Never/Sometimes/Usually/Always	135/340/321/290	78/408/322/279	92/434/330/218
**The eyes are more than 33 cm (one Chi) away from the book**			
Never/Sometimes/Usually/Always	132/373/278/300	92/467/294/228	114/534/309/118
**The finger is about 3.3 cm (one Cun) away from the tip of the nose**			
Never/Sometimes/Usually/Always	162/218/227/473	142/287/267/386	174/331/261/306
**Does your teacher remind you that your reading and writing posture is not correct?**			
Never/Sometimes/Usually/Always	181/302/227/372	191/419/231/243	357/488/158/72
**Do your parents remind you that your reading and writing posture is not correct?**			
Never/Sometimes/Usually/Always	91/272/253/467	97/274/326/387	140/419/357/159

**Figure 1:**
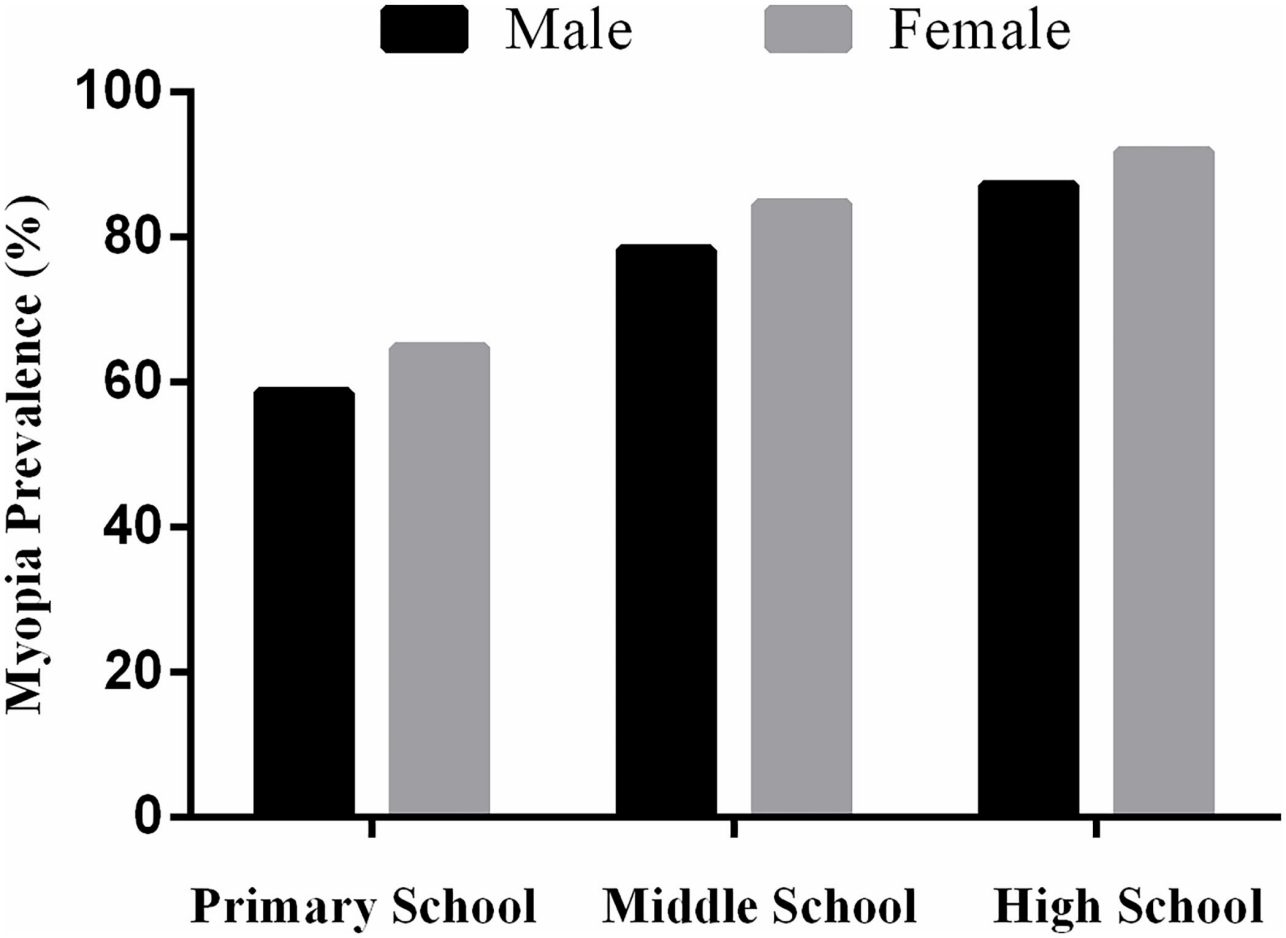
Prevalence of myopia among school students by gender.

The prevalence of myopia among primary school students was 61.49, 58.63% for male students and 64.81% for female students. The prevalence of myopia among middle school students was 81.43%, 78.28% for male students and 84.63% for female students. The prevalence of myopia among high school students was 89.72, 87.17% for male students and 91.91% for female students (Figure 1).

As seen in Table 2, after adjusting for age and grade, a reading distance of more than 33 cm was identified as a protective factor for myopia in female students. The higher the frequency of the reading distance more than 33 cm, the lower the risk of students getting myopic. Compared with female students who chose “never” for “the eyes are more than 33 cm away from the book,” the odds ratio (ORs) and 95% confidence intervals (CIs) for subjects who chose “sometimes,” “usually,” and “always” were 0.52 (0.28, 0.97), 0.49 (0.25, 0.96), and 0.31 (0.15, 0.64), respectively. In male students, keeping the finger 3.3 cm away from the nose tip was also found to be a protective factor. Compared with male students who chose “never” for “the finger is about 3.3 cm away from the tip of the nose,” the OR and 95% CI for subjects who chose “sometimes” were 0.61 (0.39, 0.96). However, keeping the chest more than the width of a fist away from the edge of the table was a risk factor for myopia in female students [usually: 1.89 (1.03, 3.49), always: 2.01 (1.04, 3.88)]. In both male and female students, the more the parents reminded them of their reading and writing postures, the higher the risk of getting myopia.

**Table 2:** Logistic regression of myopia-related factors by gender.

**Items**	**Total**		**Male**		**Female**	
	**OR (95% CI)**	***P*-value**	**OR (95% CI)**	***P*-value**	**OR (95% CI)**	***P*-value**
Age	**1.23 (1.11–1.36)**	**<0.001**	**1.21 (1.06, 1.38)**	**0.006**	**1.27 (1.09, 1.48)**	**0.002**
Grade	1.30 (0.95–1.78)	0.103	1.32 (0.87, 2.01)	0.195	1.24 (0.77, 2.01)	0.373
**The chest is more than the width of a fist from the edge of the table**						
Never	Reference					
Sometimes	1.33 (0.92–1.91)	0.126	1.27 (0.78, 2.07)	0.334	1.30 (0.73, 2.32)	0.373
Usually	**1.60 (1.09–2.35)**	**0.017**	1.27 (0.76, 2.13)	0.359	**1.89 (1.03, 3.49)**	**0.041**
Always	**1.54 (1.02–2.32)**	**0.041**	1.16 (0.67, 1.99)	0.598	**2.01 (1.04, 3.88)**	**0.039**
**The eyes are more than 33 cm (one Chi) away from the book**						
Never	Reference					
Sometimes	0.98 (0.67–1.43)	0.924	1.51 (0.92, 2.49)	0.103	**0.52 (0.28, 0.97)**	**0.041**
Usually	0.92 (0.62–1.39)	0.705	1.49 (0.86, 2.58)	0.152	**0.49 (0.25, 0.96)**	**0.036**
Always	**0.57 (0.37–0.87)**	**0.010**	0.93 (0.52, 1.64)	0.790	**0.31 (0.15, 0.64)**	**0.001**
**The finger is about 3.3 cm (one Cun) away from the tip of the nose**						
Never	Reference					
Sometimes	0.76 (0.55–1.05)	0.093	**0.61 (0.39, 0.96)**	**0.033**	1.01 (0.62, 1.65)	0.967
Usually	0.72 (0.52–1.01)	0.058	0.78 (0.49, 1.25)	0.303	0.67 (0.41, 1.09)	0.110
Always	0.90 (0.65–1.25)	0.539	0.87 (0.55, 1.37)	0.550	0.93 (0.57, 1.49)	0.748
**Does your teacher remind you that your reading and writing posture is not correct?**						
Never	Reference					
Sometimes	1.00 (0.77–1.31)	0.987	1.03 (0.71, 1.50)	0.882	1.01 (0.68, 1.51)	0.959
Usually	0.78 (0.57–1.08)	0.138	0.81 (0.52, 1.27)	0.353	0.84 (0.52, 1.36)	0.478
Always	0.72 (0.50–1.03)	0.074	0.62 (0.37, 1.03)	0.064	0.92 (0.54, 1.57)	0.757
**Do your parents remind you that your reading and writing posture is not correct?**						
Never	Reference					
Sometimes	1.25 (0.89–1.77)	0.195	1.11 (0.70, 1.74)	0.666	1.43 (0.84, 2.44)	0.189
Usually	**1.58 (1.09–2.29)**	**0.016**	1.22 (0.74, 2.00)	0.440	**2.03 (1.14, 3.61)**	**0.016**
Always	**2.10 (1.41–3.13)**	**<0.001**	**2.22 (1.28, 3.87)**	**0.005**	**1.90 (1.05, 3.45)**	**0.034**

OR, odds ratio; 95% CI, 95% confidence interval.

Adjusted for age and grade. Bold numbers are statistically significant at P < 0.05.

The associations between reading and writing postures and myopia in different school types were similar (Table 3). After adjusting for gender and age, reading distances more than 33 cm were identified as a protective factor for myopia in both primary [always: 0.55 (0.30, 0.99)] and middle school students [always: 0.37 (0.15, 0.90)]. Compared with middle school students who chose “never” for “the finger is about 3.3 cm away from the tip of the nose,” the OR and 95% CI for subjects who chose “sometimes” was 0.41 (0.21, 0.79). Still, keeping the chest more than the width of the fist away from the edge of the table was a risk factor in middle school students, and parents who were reminded of reading and writing postures were considered a risk factor in primary school students.

**Table 3:** Logistic regression of myopia-related factors by school type.

**Items**	**Primary School**		**Middle School**		**High School**	
	**OR (95% CI)**	***P*-value**	**OR (95% CI)**	***P*-value**	**OR (95% CI)**	***P*-value**
Gender	**1.32 (1.02, 1.71)**	**0.034**	1.33 (0.96, 1.84)	0.084	1.50 (0.99, 2.28)	0.059
Age	**1.55 (1.33, 1.80)**	**<0.001**	1.15 (0.97, 1.38)	0.111	0.82 (0.65, 1.03)	0.083
**The chest is more than the width of a fist from the edge of the table**						
Never	Reference					
Sometimes	1.25 (0.76, 2.05)	0.376	1.93 (0.95, 3.94)	0.070	0.50 (0.17, 1.47)	0.206
Usually	1.54 (0.91, 2.60)	0.107	**3.60 (1.66, 7.80)**	**0.001**	0.37 (0.12, 1.09)	0.072
Always	1.70 (0.97, 3.00)	0.065	**2.79 (1.25, 6.26)**	**0.013**	0.33 (0.11, 1.02)	0.054
**The eyes are more than 33 cm (one Chi) away from the book**						
Never	Reference					
Sometimes	0.78 (0.46, 1.30)	0.339	1.02 (0.47, 2.20)	0.970	1.86 (0.81, 4.26)	0.143
Usually	0.97 (0.55, 1.71)	0.913	0.58 (0.25, 1.31)	0.187	1.80 (0.74, 4.40)	0.198
Always	**0.55 (0.30, 0.99)**	**0.046**	**0.37 (0.15, 0.90)**	**0.028**	1.17 (0.43, 3.22)	0.762
**The finger is about 3.3 cm (one Cun) away from the tip of the nose**						
Never	Reference					
Sometimes	1.00 (0.63, 1.60)	0.996	**0.41 (0.21, 0.79)**	**0.008**	0.86 (0.42, 1.75)	0.666
Usually	0.72 (0.45, 1.16)	0.181	0.63 (0.32, 1.25)	0.187	0.79 (0.38, 1.67)	0.536
Always	0.96 (0.61, 1.51)	0.858	0.66 (0.34, 1.29)	0.225	1.23 (0.59, 2.58)	0.578
**Does your teacher remind you that your reading and writing posture is not correct?**						
Never	Reference					
Sometimes	0.93 (0.60, 1.42)	0.731	1.03 (0.62, 1.71)	0.921	1.15 (0.68, 1.94)	0.599
Usually	0.89 (0.54, 1.44)	0.625	0.86 (0.47, 1.58)	0.634	0.55 (0.28, 1.09)	0.085
Always	0.73 (0.44, 1.22)	0.231	0.93 (0.47, 1.82)	0.820	0.65 (0.20, 2.08)	0.464
**Do your parents remind you that your reading and writing posture is not correct?**						
Never	Reference					
Sometimes	1.31 (0.76, 2.24)	0.328	1.34 (0.71, 2.51)	0.369	0.91 (0.46, 1.76)	0.770
Usually	1.56 (0.87, 2.80)	0.137	1.39 (0.71, 2.72)	0.343	1.52 (0.72, 3.18)	0.271
Always	**1.96 (1.07, 3.57)**	**0.029**	1.91 (0.93, 3.92)	0.077	2.49 (0.88, 7.08)	0.087

OR, odds ratio; 95% CI, 95% confidence interval.

Adjusted for gender and age. Bold numbers are statistically significant at P < 0.05.

## Discussion

The prevalence of myopia among school students in our study was comparable to figures reported from other provinces and cities in China. A study covering six provinces in China found that the prevalence of myopia among primary and middle school students was 55.7%, of which the prevalence was 35.8% in the age group 6–8 years, 58.9% in the age group 10–12 years, 73.4% in the age group 13–15 years, and 81.2% in the age group 16–18 years (13). Compared with school students in other countries, the prevalence of myopia in our study population was considerably higher. The Ireland Eye Study examined 1,626 participants, and the prevalence of myopia among participants aged 6–7 years and aged 12–13 years was 3.3 and 19.9%, respectively (14). Jorge et al. revealed that the prevalence of myopia in first-year university students in Portugal was only 23.4% (15).

Reading and writing postures can affect the pleasure and effectiveness of reading and writing as well as retinal image quality, convergence and accommodation demands, and binocular comfort during the process (16). Through these factors, some investigators also considered that reading and writing postures may be an important factor in the development of myopia (17). The Myopia Investigation Study in Taipei was a population-based cohort study that followed 9–11-year-old children (*n* = 10,743) for 2 years (18). After adjustment for gender and high parental myopia, students with a near-work distance >30 cm and who discontinued near work every 30 min had significantly less myopic progression. These factors remained significant after adjusting for other behavior, suggesting that they are independent risk factors. The findings are in accordance with those reported by Ip et al. (12), who similarly found that longer reading time for pleasure and a closer reading distance (<30 cm) were associated with the progression of myopia after multivariate adjustment (*p* < 0.05 for both).

In our study, we also identified that keeping the eyes more than 33 cm away from the book and keeping the finger 3.3 cm away from the tip of the nose were protective factors for myopia in school students. Our findings were nearly consistent with previous studies. Bao et al. investigated 120 children with myopia aged 6–13 years and found that working distance decreased significantly across time for the reading and writing tasks (*p* < 0.001), suggesting that close working distance may be a risk factor for myopia progression (19). In the study by Wu et al., 4,677 students aged 16–18 years participated, and multiple logistic regression analysis showed that a higher prevalence of myopia was associated with a longer time spent for near work (OR = 1.43, 95% CI: 1.06–1.93) and shorter near-work distance (OR = 1.87, 95% CI: 1.55–2.26) (20). However, reading behavior is not a fixed entity but differs in terms of grade level and reading conditions, which also suggests that reading behavior can be altered through better ergonomics and text design that may reduce myopia and help school students to read better (21).

However, keeping the chest more than a fist away from the edge of the table and parents who were reminded of reading and writing postures were identified as risk factors for myopia, which were contrary to our common sense. Considering that once school students become myopic, their parents may pay more attention to their children's reading and writing postures and set more reminders, which may cause the prevalence–incidence bias. As for the chest-to-table distance, the results remained counterintuitive, so we cannot exclude the potential that school students may have misunderstood the question or that maintaining the chest-to-table distance can cause other changes in reading and writing postures, so we will consider further refining the questionnaires and verifying them in a larger population sample.

This study has some limitations. First, we mainly explored the associations between reading and writing postures and myopia, and there were still certain other myopia-related factors that we did not include in our study, like short-distance reading time. Second, there was recall bias and prevalence-incidence bias in our study due to the study design. Third, the feedback and perception in the three categories of students would be highly variable due to the evolved level of understanding of the questionnaire. Fourth, non-cycloplegic measurements of myopia were used, and the prevalence of myopia may have been overestimated. Last but not the least, the sample size is limited in our study and the results need to be verified in a larger population in the future.

In conclusion, maintaining an appropriate distance (>33 cm) between the eyes and the book may be good for the prevention and control of myopia.

## Data availability statement

The raw data supporting the conclusions of this article will be made available by the authors, without undue reservation.

## Ethics statement

The studies involving human participants were reviewed and approved by Ethics Committee of Ningbo Municipal Center for Disease Control and Prevention. Written informed consent to participate in this study was provided by the participants' legal guardian/next of kin.

## Author contributions

Study design: DJ and YZ. Data acquisition: HG, YG, and SZ. Statistical analysis: DJ and BS. Manuscript preparation and editing: DJ. Manuscript revision/review: YZ. All authors contributed to the article and approved the submitted version.

## Funding

This study was supported by the Ningbo Medical Science and Technology Project (No. 2021Y26), the Ningbo Science and Technology Program for Public Interest (No. 202002N3187), and the Ningbo Health Branding Subject Fund (No. PPXK2018-10).

## Conflict of interest

The authors declare that the research was conducted in the absence of any commercial or financial relationships that could be construed as a potential conflict of interest.

## Publisher's note

All claims expressed in this article are solely those of the authors and do not necessarily represent those of their affiliated organizations, or those of the publisher, the editors and the reviewers. Any product that may be evaluated in this article, or claim that may be made by its manufacturer, is not guaranteed or endorsed by the publisher.
